# Author's response—II

**Published:** 2006

**Authors:** Amit Ranjan Basu

**Affiliations:** * Independent Researcher and Doctoral Candidate Centre for Historical Studies, Jawaharlal Nehru University, New Delhi, India; *mailing address:* BE 318, Salt Lake, Kolkata 700064, West Bengal; e-mail: amitrbasu53@gmail.com

Sir,

I thank Dr Debashis Chatterjee for taking note of my article, commentaries, and offering a critique. When I appreciate his corrections of faulty historiography by Sharma, I am somewhat cautious in labelling Laing, Szasz and Cooper in such reductionist terms. A recent book has done some good review of the antipsychiatry discourse, which helps us to see this movement in context.^1^

In response to his critique on my article, first I would like to say that I have not used ‘History of Psychiatry in India’ (p.126, para 1, line 1) and the ‘History of Indian Psychiatry’ (p.126, para 2, line 1) interchangeably. In the first use, a dominant narrative strategy and its interpretation is in question and in the second, the representation of ‘Indian psychiatry’ is in question.

The concept of lack is more a psychological construct than a checklist. It is generated from the engagement between the coloniser and the colonised. It is used in relation to the hegemonic discourse of a linear, progessivist development.

I have brought Foucault to understand the disjuncture that has come in the evolutionary historiography of psychiatry and think it was irrelevant to discuss the Foucault-Derrida debate, which I have mentioned elsewhere (see References 3 and 4 in my ‘Response’). However, Derrida's critique was with the issue of madness in itself and Foucault's response to that is well acknowledged.

Finally, I appreciate Dr Chatterjee's attitude for engaging himself in the debate to make it more productive.
